# Rerouting of NADPH synthetic pathways for increased protopanaxadiol production in *Saccharomyces cerevisiae*

**DOI:** 10.1038/s41598-018-34210-3

**Published:** 2018-10-25

**Authors:** Jae-Eung Kim, In-Seung Jang, Bong Hyun Sung, Sun Chang Kim, Ju Young Lee

**Affiliations:** 1 Center for Bio-based Chemistry, Korea Research Institute of Chemical Technology (KRICT), 406-30, Jongga-ro, Jung-gu, Ulsan 44429 Republic of Korea; 2 Cell Factory Research Center, Korea Research Institute of Bioscience and Biotechnology (KRIBB), Daejeon, 34141 Republic of Korea; 3 Department of Biological Sciences, Korea Advanced Institute of Science and Technology (KAIST), Daejeon, 34141 Republic of Korea

## Abstract

Ginseng (*Panax ginseng*) and its bioactive components, ginsenosides, are popular medicinal herbal products, exhibiting various pharmacological effects. Despite their advocated use for medication, the long cultivation periods of ginseng roots and their low ginsenoside content prevent mass production of this compound. Yeast *Saccharomyces cerevisiae* was engineered for production of protopanaxadiol (PPD), a type of aglycone characterizing ginsenoside. PPD-producing yeast cell factory was further engineered by obtaining a balance between enzyme expressions and altering cofactor availability. Different combinations of promoters (*P*_*GPD*_, *P*_*CCW12*_, and *P*_*ADH2*_) were utilized to construct the PPD biosynthetic pathway. Rerouting the redox metabolism to improve NADPH availability in the engineered *S*. *cerevisiae* also increased PPD production. Combining these approaches resulted in more than an 11-fold increase in PPD titer over the initially constructed strain. The series of metabolic engineering strategies of this study provides a feasible approach for the microbial production of PPD and development of microbial platforms producing other industrially-relevant terpenoids.

## Introduction

Ginseng (*Panax ginseng*) is one of the most widely used medicinal herbs in Asia, Europe, and North America to alleviate fatigue, enhance immunity, and improve physical performance. Representing the major bioactive components among various constituents of ginseng, ginsenosides are a group of triterpenoids exhibiting diverse pharmacological effects, especially in antitumor therapy and neurological function recovery^1–5^. Ginsenosides are traditionally extracted from ginseng roots that contain at least 4% ginsenosides by dry weight^6^. Wild ginseng roots are, however, rarely available for the mass production of ginsenosides. Although the current market supply mainly relies on the cultivation of ginseng in fields, the process of growing ginseng roots from seed to harvest for extraction is time-consuming and labor-intensive. Cultivation of ginseng also requires extensive effort, since its growth is reliant upon optimizing many environmental factors, such as soil, shade, climate, pathogens, and pests^7^.

Microorganisms, such as *Escherichia coli* and *Saccharomyces cerevisiae*, provide alternative and attractive ways of producing diverse natural chemicals from renewable resources. Since microbial metabolism is not always a viable method of producing chemicals of interest or desired products, metabolic engineering is required to facilitate the conversion of these recalcitrant microorganisms into highly efficient and cooperative microbial cell factories^8^. As metabolic engineering and synthetic biology tools continue to improve, various microbial cell factories have been engineered to efficiently manufacture valuable natural products^9–13^.

**Figure 1:**
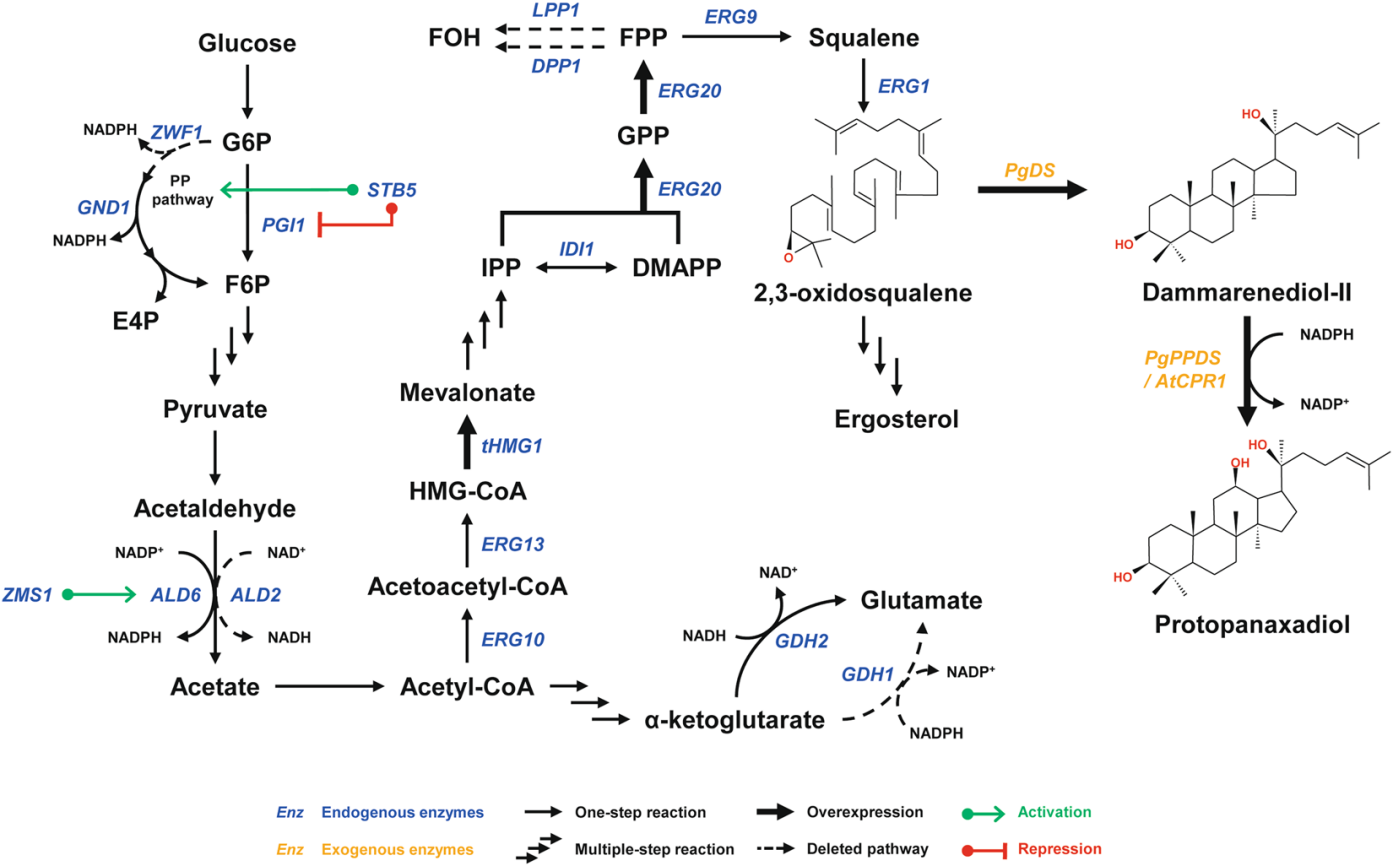
Biosynthetic pathway for protopanaxadiol (PPD) production in metabolically engineered *S*. *cerevisiae*. Exogenous plant genes, *PgDS*, *PgPPDS*, and *AtCPR1*, were introduced to construct the biosynthetic pathway for PPD synthesis. The native yeast pathways for farnesol (FOH) production by *LPP1* and *DPP1* were deleted with the overexpression of *tHMG1* and *ERG20* for enhancement of the precursor supply. Genetic targets identified for improvement of NADPH availability were manipulated to increase the production of PPD. Single arrows represent one-step enzymatic conversions, while triple arrows represent multiple reactions. Bold arrows represent overexpressed genes. Dashed arrows represent deleted reactions.

Recently, a microbial platform was developed through the metabolic engineering of *S*. *cerevisiae* for the efficient production of protopanaxadiol (PPD), a type of aglycone that characterizes certain ginsenosides^9^. PPD itself can be further converted to ginsenosides by various glycosyltransferases (GTs), serving as the basis for the microbial production of ginsenosides. All eukaryotic cells (including plant cells) utilize the same mevalonic acid (MVA) pathway to produce two common building blocks, isopentenyl pyrophosphate (IPP) and dimethylallyl pyrophosphate (DMAAP), for the biosynthesis of triterpenoids (Fig. 1). In *P*. *ginseng*, 2,3-oxidosqualene is an essential precursor for PPD synthesis through cyclization and hydroxylation by dammarenediol-II synthase (PgDS) and protopanaxadiol synthase (PgPPDS), respectively^14,15^. In wild-type yeast, most 2,3-oxidosqualene enters the ergosterol synthetic pathway for cellular membrane formation. Introduction of an artificial synthetic ginsenoside pathway, consisting of PgDS and PgPPDS with *Arabidopsis thaliana* NADPH-cytochrome p450 reductase (AtCPR1), allowed the fermentative production of ginsenosides from 2,3-oxidosqualene in *S*. *cerevisiae* (Fig. 1).

The synthetic pathway for ginsenoside production in *S*. *cerevisiae* encompasses many of the challenges associated with engineering metabolic pathways to improve production titers, through enhancing the activity of related enzymes and increasing availability of precursors and relevant cofactors, while preventing severe growth inhibition. Much of the recent work on enhancing microbial production of ginsenosides has been targeted toward improving the enzymatic performance of the biosynthetic pathway by increasing the copy numbers of rate-limiting enzymes, integrating truncated 3-hydroxy-3-methylglutaryl-CoA reductase (tHMG1) to bypass feedback regulation of the MVA pathway, and constructing fusion enzymes for substrate channeling^9,16–18^. Further optimization through the application of metabolic engineering strategies and synthetic biology tools is required to enable mass production of ginsenosides at a reduced price and broader application for clinical trials and the food supplement industry.

In this study, we show that a series of metabolic engineering strategies that alter cofactor availability and redirect metabolic flux toward the synthetic ginsenoside pathway results in increased PPD production in *S*. *cerevisiae*. Firstly, the impact of promoter strength and regulation on two key enzymes, PgDS and PgPPDS, was examined to find an optimal balance between stability of expression and transcriptional strength for improved PPD production. PgDS and PgPPDS are among the heterologous enzymes for microbial production of PPD in yeast. To access whether the balanced expression levels between PgDS and PgPPDS was able to improve the metabolic flux toward the production of PPD, these two fundamental enzymes were investigated with different sets of promoters. Three well-characterized promoters, *P*_*GPD*_, *P*_*CCW12*_, and *P*_*ADH2*_, showing different carbon-source dependence and transcriptional strength, were compared in PPD-producing strains. Secondly, the cellular redox balance was engineered in favor of PPD synthesis by the provision of sufficient NADPH. The redox cofactor, NADPH, which plays a critical role in the metabolism of *S*. *cerevisiae*, is also essential for PPD biosynthesis. Although NADPH is dynamically consumed and replenished during cellular metabolism, perturbations of the redox cofactor can significantly affect the production profiles of artificial pathways consisting of heterologous redox enzymes^19–21^. The availability of NADPH was enhanced by replacing a NADH-generating enzyme (ALD2) with a functionally equivalent enzyme (ALD6) generating NADPH instead (Fig. 1). Lastly, the *zwf1* gene encoding glucose 6-phosphate dehydrogenase was identified as a genetic deletion target for its potential to improve PPD production by maximizing carbon flux toward PPD biosynthesis. By constructing a synthetic pathway coupled with redox balance engineering, we were able to achieve a final PPD production titer of 6.01 mg/L, which was more than an 11-fold improvement from that of the initially constructed strain. Furthermore, for the first time, we demonstrated that increasing the availability of NADPH improves PPD production in *S*. *cerevisiae*. This result also suggests that the engineering strategies employed in the present study are not only feasible for enhanced microbial production of PPD, but for the development of improved microbial platforms in producing other industrially-relevant terpenoids, as well.

## Results and Discussion

### Construction of protopanaxadiol synthetic pathway in *S*. *cerevisiae*

In order to increase the supply of precursors, the MVA pathway of a wild-type yeast strain (CEN.PK2-1D) was modified in a stepwise manner to secure the supply of squalene, serving as a precursor for PPD biosynthesis. The 3-hydroxy-3-methylglutaryl-CoA reductase (HMG1) is known as a rate-limiting enzyme in the MVA pathway due to the post-transcriptional feedback inhibition, and overexpression of truncated HMG1, lacking its membrane-binding region, has been reported to accumulate large amounts of squalene in yeast^22^. The *tHMG1* gene is commonly used for strain engineering to increase terpenoid production *via* the MVA pathway^22,23^. The *erg20* gene encoding farnesyl diphosphate synthase (FPS) is also essential for squalene accumulation, and its bifunctional activities, dimethylallyl transtransferase and geranyl transtransferase, appear to be rate-limiting in native yeast metabolism^24^.

**Figure 2:**
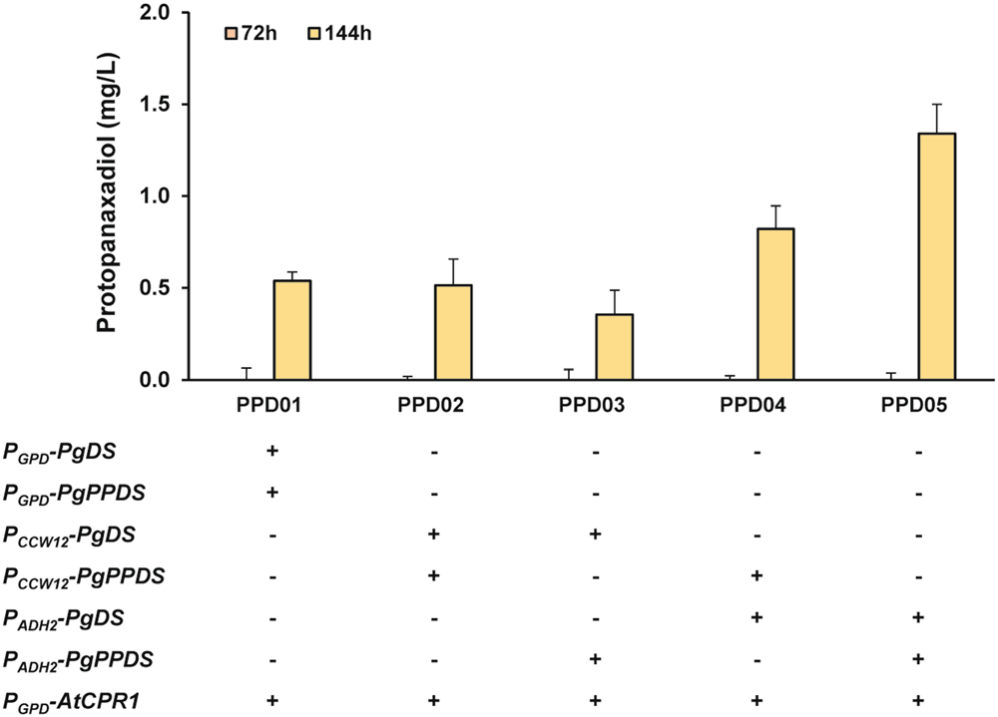
Comparison of PPD productions by engineered *S*. *cerevisiae* strains expressing PgDS and PgPPDS under different combinations of three promoters (*P*_*GPD*_, *P*_*CCW12*_, and *P*_*ADH2*_). All strains utilize the same *GPD* promoter for *AtCPR1* expression. Each strain was cultivated for 144 hours at 30 °C in 50 mL of YSC-URA medium containing 20 g/L glucose. Samples for PPD detection were withdrawn at 72 and 144 hours of cultivation. The data are the means and standard deviations from three independent experiments with at least two biological replicates.

The *tHMG1* gene (controlled by the *CCW12* promoter) and *erg20* gene (controlled by the *TEF1* promoter) were first integrated into the chromosome of the yeast at the *HIS3* and *LEU2* sites, respectively. A cytochrome P450 reductase gene from *A*. *thaliana* (*AtCPR1* controlled by the *GPD* promoter) was then integrated at the *LPP1* site, resulting in strain PPD00 (Table 1). The *P*. *ginseng* dammarenediol-II synthase gene (*PgDS* controlled by the *GPD* promoter) and the *P*. *ginseng* PPD synthase gene (*PgPPDS* controlled by the *GPD* promoter) were integrated into the chromosome of strain PPD00 at the *DPP1* and *YPL062w* sites, respectively, resulting in strain PPD01. The *LPP1* and *DPP1* genes are associated with farnesol production at the FPP branch point^25^ (Fig. 1). Thus, these sites were chosen as integration loci to minimize metabolic flux towards farnesol synthesis. The disruption of the *YPL062w* gene has previously been reported to elevate intracellular mevalonate levels^26^. Strain PPD01 produced negligible amounts of PPD at 72 hours and 0.54 mg/L of PPD at 144 hours when cells were cultivated in flasks at 30 °C (Fig. 2, Table 2).

**Table 1:** List of strains used in this study.

Strains	Genotype	Source
CEN.PK2-1D	*MATα ura3-52 trp1-289 leu2-3*,*112 his3*Δ*1 MAL2-8*^*C*^ *SUC2*	Euroscarf
−ALD2	CEN.PK2-1D Δ*ald2*	This study
−GDH1	CEN.PK2-1D Δ*gdh1*	This study
+GND1	CEN.PK2-1D, pGPD-GND1	This study
+GDH2	CEN.PK2-1D, pGPD-GDH2	This study
+ALD6	CEN.PK2-1D, pGPD-ALD6	This study
+ZWF1	CEN.PK2-1D, pGPD-ZWF1	This study
+STB5	CEN.PK2-1D, pGPD-STB5	This study
−ALD2 + ALD6	CEN.PK2-1D Δ*ald2::P*_*GPD*_*-ald6*	This study
−GDH1 + GDH2	CEN.PK2-1D Δ*gdh1::P*_*GPD*_*-gdh2*	This study
PPD00	CEN.PK2-1D Δ*leu2::P*_*TEF1*_*-ERG20* Δ*his3::P*_*CCW12*_*-tHMG1* Δ*lpp1::P*_*GPD*_*-AtCPR1*	This study
PPD01	PPD00 Δ*dpp1::P*_*GPD*_*-PgDS* Δ*ypl062w::P*_*GPD*_*-PgPPDS*	This study
PPD02	PPD00 Δ*dpp1::P*_*CCW12*_*-PgDS* Δ*ypl062w::P*_*CCW12*_*-PgPPDS*	This study
PPD03	PPD00 Δ*dpp1::P*_*CCW12*_*-PgDS* Δ*ypl062w::P*_*ADH2*_*-PgPPDS*	This study
PPD04	PPD00 Δ*dpp1::P*_*ADH2*_*-PgDS* Δ*ypl062w::P*_*CCW12*_*-PgPPDS*	This study
PPD05	PPD00 Δ*dpp1::P*_*ADH2*_*-PgDS* Δ*ypl062w::P*_*ADH2*_*-PgPPDS*	This study
PPD06	PPD05, pGPD-ZWF1	This study
PPD07	PPD05, pGPD-STB5	This study
PPD08	PPD05 Δ*ald2::P*_*GPD*_*-ald6*	This study
PPD09	PPD05 Δ*gdh1::P*_*GPD*_*-gdh2*	This study
PPD10	PPD05, pGPD-ZMS1	This study
PPD11	PPD08, pGPD-ZMS1	This study
PPD12	PPD08 Δ*zwf1*	This study

**Table 2:** Production profile of selected metabolites by engineered *S*. *cerevisiae* strains.

Time (h)	Strains	OD_600_	SQ (mg/L)	OSQ (mg/L)	DD (mg/L)	PPD (mg/L)
72	PPD01	25.90	1.02	1.31	0.98	N.D.
	PPD05	25.66	0.79	1.21	0.97	N.D.
	PPD08	14.35	0.10	0.46	0.74	1.58
	PPD10	15.23	0.87	0.80	0.90	1.67
	PPD11	11.11	0.13	0.57	0.75	0.93
	PPD12	13.81	0.00	0.42	0.88	1.17
144	PPD01	24.05	1.25	1.58	0.47	0.54
	PPD05	23.65	2.35	1.54	1.84	1.34
	PPD08	13.41	0.23	0.20	1.96	6.01
	PPD10	14.67	2.02	0.44	1.35	3.35
	PPD11	9.92	0.26	0.00	1.56	4.48
	PPD12	12.87	0.28	0.16	2.02	5.66

Note: All strains were cultivated for 6 days at 30 °C in 50 mL of YSC-URA medium containing 20 g of glucose/L. The data shown are the averages of three independent experiments. Standard deviations were less than 5%. SQ, squalene; OSQ, 2,3-oxidosqualene; DD, dammarenediol-II; PPD, protopanaxadiol; N.D., not detected.

### Characterization and selection of promoters for efficient protopanaxadiol production

A wide range of promoters is available for use in yeast, many of which are well-characterized native promoters of *S*. *cerevisiae*. The variety of promoters with different behaviors provides a useful tool for metabolic engineering. Selecting a suitable promoter is instrumental in efficient gene expression and consequent metabolite formation^27^. We examined the impact of promoter strength and balance between two key enzymes, PgDS and PgPPDS, on PPD production in yeast. PgDS and PgPPDS located in distant regions of the chromosome were expressed under different combinations of three promoters: *P*_*GPD*_, *P*_*CCW12*_, and *P*_*ADH2*_. Both *P*_*GPD*_ and *P*_*ADH2*_ are strong promoters with opposite tendencies in the presence of glucose as a carbon source; *P*_*GPD*_ tends to be highly activated by the presence of glucose and becomes reduced as the level of glucose decreases, while *P*_*ADH2*_ is strongly repressed by high glucose levels and is activated when cells switch to growth on ethanol. *P*_*CCW12*_ is a constitutive promoter measured as strong as *P*_*GPD*_ and was reported to maintain a similar level of its initial activity over all stages of growth^28,29^.

In this study, the synthetic pathway for PPD production in yeast was initially constructed with the *GPD* promoter for expressions of PgDS, PgPPDS, and AtCPR1, expecting the strong promoter to enable PPD production from the early stages of fermentation. Engineered strains with PgDS and PgPPDS under different combinations of *P*_*CCW12*_ and *P*_*ADH2*_ were constructed and evaluated for PPD production in comparison to strain PPD01 (Fig. 2). Strain PPD02, expressing both PgDS and PgPPDS under the *CCW12* promoter (*P*_*CCW12*_), produced a similar level of PPD to strain PPD01 at 144 hours of fermentation. The use of *ADH2* promoter (*P*_*ADH2*_) for the expression of either PgDS or PgPPDS resulted in opposite PPD production profiles. Strain PPD03 (expressing PgDS under the *CCW12* promoter and PgPPDS under the *ADH2* promoter) showed slightly decreased PPD production, while strain PPD04 (expressing PgDS under the *ADH2* promoter and PgPPDS under the *CCW12* promoter) showed about a 1.5-fold increase in PPD production compared with strain PPD01. When the *ADH2* promoter was adopted for both PgDS and PgPPDS, the PPD titer increased by 2.5-fold at 144 hours. The resulting strain, PPD05, produced up to 1.34 mg/L PPD at 144 hours of fermentation in flasks at 30 °C. All engineered strains produced negligible amounts of PPD at 72 hours of fermentation. The PPD05 strain was used for further engineering work in this study to improve PPD production.

Taken together with the native metabolic pathway of yeast producing ethanol during glucose-fed fermentation, the PPD production profile indicated that PPD production is closely coordinated with the ethanol-dependent growth phase of yeast. Among the engineered strains with different promoters, the highest PPD production was achieved by strain PPD05, expressing both PgDS and PgPPDS under the *ADH2* promoter. This strong, inducible promoter enabled the yeast to efficiently control the synthetic pathway by separating the PPD production phase from the cell growth phase. In contrast, PPD production by strains PPD01 and PPD02 was under metabolic competition with cell growth, resulting in low PPD titers. Greater decreases in PPD production titers of strain PPD03 were likely due to accumulation of the potentially toxic intermediate, dammarenediol-II, by the imbalanced regulation of promoters. Comparing with strain PPD03, the increased PPD production in strain PPD04 also showed that the phase separation is important in PPD production. The dynamic control system using carbon source-dependent promoters was previously used to optimize target pathways and increase production titers^10^. This data indicate that different characteristics of promoters in strength and regulation patterns play a significant role in optimizing synthetic pathways in heterologous hosts.

### Engineering the redox balance to enhance protopanaxadiol production

Engineering redox cofactor metabolism has been a major focus in metabolic engineering to increase product yields in microbial processes. The synthetic pathway for PPD production in yeast involves reactions of redox enzymes, such as tHMG1, ERG9, and PgPPDS, consuming NADPH as an essential cofactor. We, therefore, manipulated multiple genes involved in biosynthesis or utilization of NADPH to redistribute cytosolic NADPH for the enhanced production of PPD. Genetic targets to explore for the redox balance engineering were *ald2*, *ald6*, and *zms1* in the acetate pathway, *gdh1* and *gdh2* in the ammonium assimilation pathway, and *zwf1*, *gnd1*, and *stb5* in the pentose phosphate (PP) pathway (Fig. 1). All these target genes encode proteins involved in biosynthesis of NAD(P)H by either directly consuming or generating cofactors, or indirectly affecting others as a transcription factor. The *ald2* gene encodes cytoplasmic aldehyde dehydrogenase utilizing NAD^+^ as the preferred cofactor to convert acetaldehyde to acetate. The *ald6* gene encodes the NADP^+^-preferred isozyme of ALD2. The *gdh1* gene encodes NADP^+^-dependent glutamate dehydrogenase, which consumes a substantial amount of NADPH in the cytosol, while the *gdh2* gene encodes NAD-dependent isozyme. The *gnd1* and *zwf1* genes encode 6-phosphogluconate dehydrogenase and glucose 6-phosphate dehydrogenase, respectively, for the PP pathway, thus providing the major source of NADPH required for reductive biosynthetic reactions in yeast. The *stb5* gene encodes a transcription factor involved in multiple gene regulations related to the oxidative stress response. As a zinc cluster protein, STB5 has been reported to be a basal regulator of the PP pathway^30^. It was speculated that alteration of cofactor dependence by replacing either a NADH-generating enzyme to a NADPH-generating enzyme, or a NADPH-consuming enzyme to a NADH-consuming enzyme would be able to enhance the availability of NADPH for PPD production.

**Figure 3:**
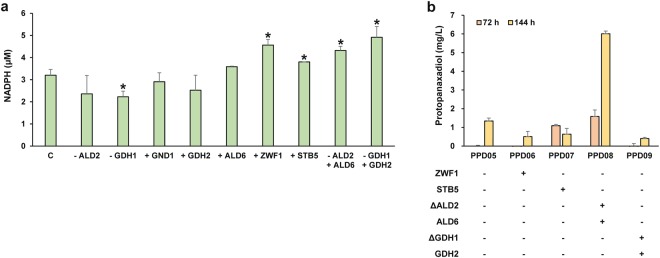
Engineering NADPH availability for increased PPD production. (**a**) NADPH concentration after a 24-hour cultivation of the wild-type and constructed strains with genetic modification in pathways for the redox cofactor metabolism. (**b**) PPD titers (mg/L) in engineered strains with selectively applied genetic modifications for increased PPD production. All strains were cultivated at 30 °C in 50 mL of YSC-URA medium containing 20 g/L glucose. For the wild-type strain, uracil was supplemented at 20 μg/mL. The data are the means and standard deviations from three independent experiments with at least two biological replicates. C, wild-type CEN.PK2-1D control strain. An asterisk (*) indicates that the value is significantly different (*P* < 0.05) from the control strain, C.

The impact of engineering the redox balance on PPD production was primarily characterized by measuring cytosolic NADPH concentrations. CEN.PK2-1D was used as a parent strain to construct various engineered strains with genetic modification to the NADPH biosynthetic pathway. Among the target genes above, those encoding NADPH-consuming enzymes were deleted or, if encoding NADPH-generating enzymes, overexpressed. The NADPH concentration was measured at 24 hours of flask fermentation. Compared with CEN.PK2-1D, engineered strains with deletion of either the *ald2* or *gdh1* gene, or overexpression of either the *gnd1* or *gdh2* gene showed decreased NADPH concentrations (Fig. 3a). When the *ald6* gene was overexpressed, a similar level of NADPH was measured as was seen in the wild-type yeast strain. Overexpression of the *zwf1* or *stb5* gene increased NADPH concentrations by 1.4- and 1.2-fold, respectively, confirming that the PP pathway provides a significant source of NADPH. The mutant strains engineered with a single target gene showed a negligible effect (as in + ALD6 strain) or even adverse effects (as in -ALD2, -GDH1, + GND1, and + GDH2 strains) on NADPH accumulation. Therefore, isoforms of the target genes were simultaneously engineered to compensate their metabolic activity while altering utilization of the preferred cofactor. ALD6 overexpression with deletion of ALD2 (-ALD2 + ALD6) and GDH2 overexpression with deletion of GDH1 (-GDH1 + GDH2) resulted in increased NADPH concentrations by 1.3- and 1.5-fold, respectively, compared with the wild-type CEN.PK2-1D strain (Fig. 3a).

Based on the results of the redox balance engineering, positively-influencing target genes were selected to apply toward PPD production in strain PPD05. All engineered strains resulted in decreased PPD production compared with PPD05, except PPD08 (Fig. 3b). Deletion of ALD2 coupled with ALD6 overexpression provided approximately a 4.5-fold improvement in PPD production compared with strain PPD05. None of the other target genes (+ZWF1 and +STB5) or a combination of genes (−GDH1 +GDH2) shown to increase NADPH concentrations led to an increase in PPD production. Strain PPD08 produced up to 1.58 mg/L at 72 hours and 6.01 mg/L at 144 hours of fermentation (Fig. 3b).

Compared with NADH, NADPH is more tightly regulated in the cell, as this cofactor is essential for the biosynthesis of cellular macromolecules^31,32^. NADPH is also a major cofactor for the synthetic PPD production pathway in yeast. Increased PPD production in strain PPD08 can be correlated with an increased cytosolic NADPH concentration by shifting the cofactor preference in the acetate pathway. Overexpression of ZWF1 or STB5 increased cytosolic NADPH concentrations, but resulted in decreased PPD production. ZWF1 catalyzes the first step of the PP pathway which competes with the glycolysis pathway for limited glucose 6-phosphate. STB5 has been reported, not only to upregulate the expression of most genes in the PP pathway, but also to repress the expression of PGI1, phosphoglucose isomerase, driving glucose 6-phosphate toward the glycolysis pathway over the PP pathway^31,33^. Thus, overexpression of ZWF1 or STB5 could constrain the utilization of carbon fluxes toward acetyl-CoA formation, resulting in low PPD production. Overexpression of GDH2 with deletion of GDH1 for PPD production was not effective as shown in strain PPD09, resulting in even lowered PPD titer than strain PPD05. It seemed the carbon flux into PPD production was also constrained in the strain PPD09. GDH2 normally degrades glutamate to α-ketoglutarate and ammonia, but the overexpression of this enzyme can promote catalysis of the reverse reaction, glutamate biosynthesis, which is normally executed by GDH1^34^. Deletion of GDH1, therefore, could be compensated with GDH2 overexpression. Such genetic swapping was expected to increase PPD production through the reduced use of NADPH for glutamate biosynthesis. As in the case of ZWF1 and STB5, GDH2 overexpression coupled with the deletion of GDH1 enabled to increase NADPH concentration; strain −GDH1 +GDH2 achieved the highest NADPH concentration among all engineered strains constructed in this study, but the excess NADPH was not used for PPD production. We speculated that carbon fluxes after acetyl-CoA formation overflowed into glutamate biosynthesis, due to the overexpressed GDH2. Along with the overexpressed target genes of *zwf1* and *stb5* in PPD06 and PPD07 strains respectively, the overexpression of GDH2 with deletion of GDH1 could only increase the cofactor concentration, but not PPD titers. All these targets genes were engineered in favor of producing more NADPH, but it was speculated that they also created more metabolic competition of the carbon flux against PPD production. These results suggest that excess cofactors can lead to an improved production profile only when the carbon flux is maintained adequately for the synthetic PPD production pathway.

### Increased protopanaxadiol production by the enhancement of NADPH/NADP^+^ ratio

**Figure 4:**
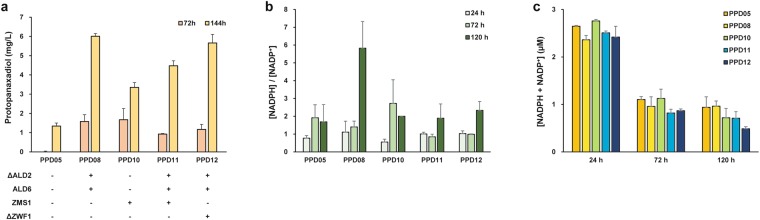
PPD production, NADPH/NADP^+^ ratios and total amounts of NADPH + NADP^+^ by the engineered *S*. *cerevisiae* strains. All strains were cultivated for 6 days at 30 °C in 50 mL of YSC-URA medium containing 20 g/L glucose. (**a**) PPD titer (mg/L); (**b**) cytosolic NADPH/NADP^+^ ratio; (**c**) total concentration of NADPH + NADP^+^. Samples for PPD production were withdrawn at 72 and 144 hours of cultivation, while samples for NADPH and NADP^+^ measurement were withdrawn at 24, 72, and 120 hours of cultivation. The data are the means and standard deviations from three independent experiments with at least two biological replicates.

The major enzymatic source of NADPH generation is the reaction catalyzed by glucose 6-phosphate dehydrogenase, encoded by the *zwf1* gene, of the PP pathway. Accordingly, disruption of the *zwf1* gene triggers NADPH starvation, resulting in significant growth reduction, methionine auxotrophy, and increased sensitivity to oxidizing agents^32^. In an effort to clarify alternative routes for NADPH synthesis, three highly homologous isocitrate dehydrogenases, IDP1, IDP2, and IDP3, were presumed to play a particular role with ZWF1, but single, double, or triple deletions of these isozymes in the *zwf1*-null mutant produced minor or inconsequential growth deficiencies, suggesting that their enzymatic contributions to NADPH generation are limited^35^. In contrast, deletion of the *ald6* gene was found to be lethal to cell viability with the disruption to the *zwf1* gene, indicating that the reaction catalyzed by ALD6 is an important source of NADPH generation in yeast^32^. This finding elucidates our result that overexpression of ALD6 slightly increased the NADPH concentration, and the same overexpression in place of ALD2 further improved NADPH generation (Fig. 3), leading to increased PPD production by the engineered PPD08 strain (Fig. 4a, Table 2).

In addition to the *ald6* gene, overexpression of the *zms1* gene encoding a zinc cluster protein was reported to restore a severe growth deficiency exhibited by the *zwf1*-null mutant strain^32^. Overexpression of the *zms1* gene increased the *ald6* gene transcript level. As a transcription factor up-regulating the *ald6* gene, it was justifiable to expect that the overexpression of ZMS1 would improve PPD production through enhancement of NADPH generation. To verify this hypothesis, the *zms1* gene was overexpressed in strain PPD05, generating strain PPD10. Strain PPD10 produced 1.67 mg/L of PPD at 72 hours and 3.35 mg/L of PPD at 144 hours, which represents a 2.5-fold increase in titers relative to strain PPD05 (Fig. 4a, Table 2). The overall PPD titers of strain PPD10, however, were lower than those of PPD08. This was likely because ALD6 was more potent than ZMS1 at the enzymatic level of regulation on NADPH generation. This result is compatible with a previous study showing that ALD6 is more influential than ZMS1 in suppressing deficient growth effects of the *zwf1*-null strain^32^. In addition, the *zms1* gene was overexpressed in strain PPD08, generating strain PPD11. However, ZMS1 overexpression in combination with ALD6 overexpression did not allow further improvement in PPD production, instead decreased PPD production due to concomitant growth inhibition (Fig. 4a, Table 2). The *zms1* gene product was reported to influence the expression of mitochondria- and respiration-related nuclear genes in yeast^36^. Thus, the overexpression of the *zms1* gene was likely a substantial metabolic burden to the cells in which a redox balance is already re-shaped for heterologous biosynthesis.

As the first step of the PP pathway playing a critical role in the cytosolic redox balance, ZWF1 has been a frequent target gene for overexpression to increase availability of NADPH or meet additional demand for NADPH imposed by target metabolite synthesis. Overexpression of ZWF1 has been employed for terpenoid production in *S*. *cerevisiae*, such as alkaloid strictosidine^37^, carotenoid^38^, and squalene^23^. Its overexpression has also been proven to improve lysine production in *Corynebacterium glutamicum*^39^ and xylitol production in *S*. *cerevisiae*^40^. In our case, overexpression of ZWF1 improved NADPH generation, but did not lead to an increase in PPD production (Fig. 3). In addition, the disruption of the PP pathway was previously reported to increase carbon fluxes through cytosolic aldehyde dehydrogenase in the acetate production pathway^11,41^. Therefore, we chose to delete ZWF1 for the elimination of the PP pathway. The increase in flux through ALD6 was expected to promote the NADP^+^-dependent conversion of acetaldehyde to acetate, rendering this pathway a new major source of NADPH for the cell. When the *zwf1* gene was deleted in strain PPD08, the resulting strain PPD12 showed similar levels of PPD production to the parent strain (PPD08) (Fig. 4a, Table 2).

To evaluate the effect of NADPH availability for enhancement of PPD production, both NADPH/NADP^+^ ratios and total levels of NADPH and NADP^+^ ([NADPH + NADP^+^]) were determined (Fig. 4b,c). The measurements were carried out at three different time points: 24, 72, and 120 hours of fermentation. The NADPH/NADP^+^ ratios showed a good accordance with the variation of PPD production by engineered strains (Fig. 4a,b). This suggests that higher PPD production might be ascribed to an increase in NADPH availability and improved cofactor ratios, since the synthetic PPD production pathway requires NADPH by means of several enzymes, especially PgPPDS with AtCPR1. All engineered strains showed similar levels of total concentrations of NADPH and NADP^+^ at 24 hours (Fig. 4c). By 72 hours, overall concentrations decreased to less than half of the concentration values measured at 24 hours, as a result of the growth cycle entering the stationary phase (the initial production phase for PPD synthesis). After 72 hours, the total concentrations of NADPH and NADP^+^ remained fairly constant over 120 hours of fermentation, except for strain PPD12 (Δ*zwf1* strain). The deletion of the *zwf1* gene caused a nearly 50% decline in total concentrations of NADPH and NADP^+^ from its parent strain (PPD08) at 120 hours (Fig. 4c). Considering that the total concentrations of NADPH and NADP^+^ from the PPD08 and PPD12 strains were similar at 24 and 72 hours, the 50% reduction at 120 hours would be significant enough to decrease available NADPH for PPD production. The decreased availability of NADPH could have substantially disturbed the PPD production pathway. Strain PPD12, however, exhibited a similar level of PPD production to strain PPD08, implying the effect of carbon fluxes redirected to enhance the PPD production pathway.

**Figure 5:**
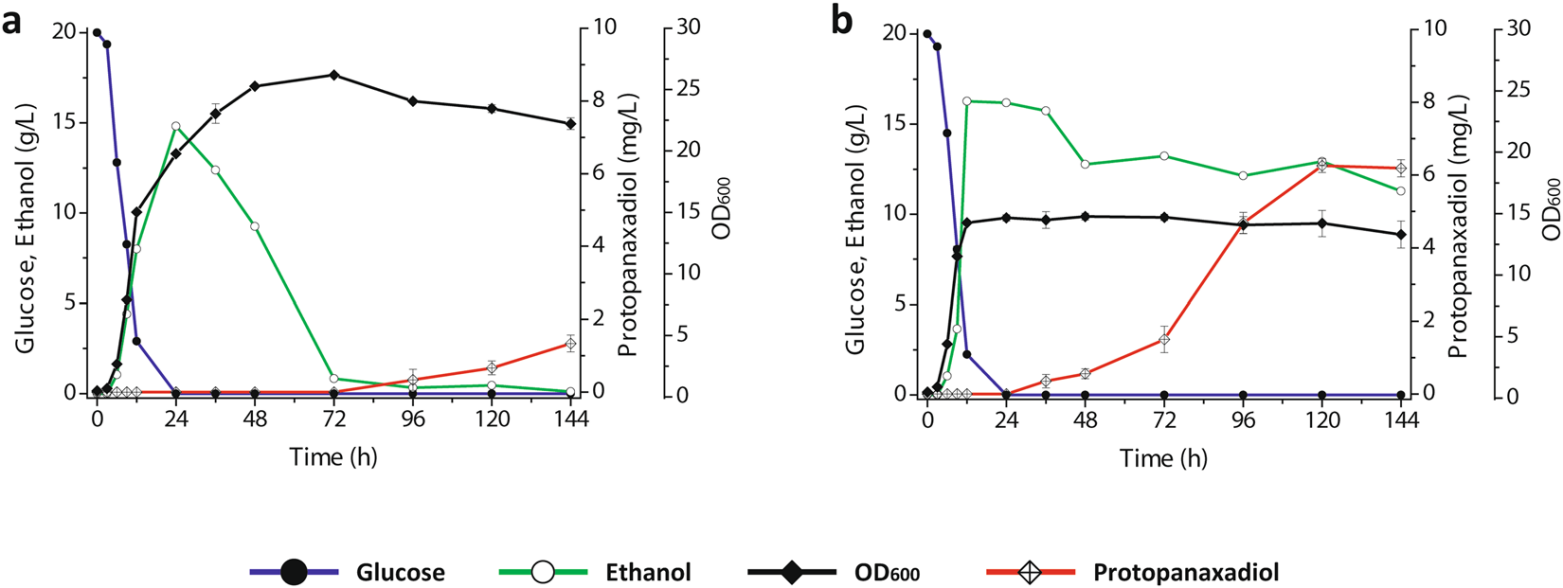
Growth, consumption of carbon sources, and PPD production profiles of the *S*. *cerevisiae* strains with different genetic modifications. (**a**) strain PPD05 (PPD00 Δ*dpp1*::*P*_*ADH2*_*-PgDS* Δ*ypl062w*::*P*_*ADH2*_*-PgPPDS*); (**b**) strain PPD08 (PPD05 Δ*ald2*::*P*_*GPD*_*-ald6*). All strains were grown at 30 °C in 50 mL of YSC-URA medium containing 20 g/L glucose with an initial OD_600_ of 0.5. The data display experiments performed from a set of two independent duplicate cultures for each strain; the error bars represent standard deviations.

Production profiles of precursor metabolites and cell density from these engineered strains were provided in Table 2. In addition, time-course profiles of PPD production and substrate consumption for the optimized strain PPD08 were analyzed (Fig. 5). All the engineered strains for improvement on redox balance showed growth defects when compared to the PPD01 and PPD05 strains (Table 2). This result was predictable to some extent, as the redox cofactors are among the highly connected molecules in metabolic networks and thus redox homeostasis is tightly regulated as a fundamental requirement for sustained metabolism and growth in yeast^42^. NADH is mostly associated with the catabolism of glucose for energy generation, while NADPH is used for anabolic reactions. These cofactors together are involved in several hundred enzymatic reactions in yeast^43,44^. Such ubiquitous role of redox cofactors has been an engineering target to bring far-reaching effects on fermentation kinetics and production profiles from central carbon metabolism^42^. This suggests that small perturbations on redox balance required enough to improve heterologous pathways, although seemingly negligible compared to alterations in biomass, could result in noticeable growth deficiency. In this study, our engineered strains also exhibited growth defects, yet the strategy of rerouting NADPH synthetic flux for PPD production increased PPD titers from the engineered strains (Fig. 5). Comparing to PPD05 strain, PPD08 strain exhibited much steeper pattern of entering the stationary phase of growth which results in reduced cell mass and slow utilization of ethanol (Fig. 5). The engineered strain utilizing ethanol slowly would be able to use the carbon source more efficiently with excess NADPH for PPD production. This result implies again that the PPD production is closely coordinated with the ethanol-dependent growth phase of yeast.

Besides the genes of investigation in this study, glucose 6-phosphate dehydrogenase could have been a good candidate for overexpression when considering the direction of its catalytic activity is in accordance with the metabolic flux toward PPD production. As a potential strategy to increase the NADPH/NADP^+^ ratio, we have also overexpressed an independent cofactor regenerating enzyme, NADH kinase encoded by the *pos5* gene. However overexpression of POS5 stunted cell growth, resulting in extremely low PPD production (data not shown). Therefore, along with the redox metabolism engineering, the future work on metabolic engineering of *S*. *cerevisiae* for ginsenoside production will be necessary to combine the previous works together, which include enhancement of the heterologous ginsenoside pathway and optimization of fermentative process for mass production of ginsenoside in *S*. *cerevisiae*, with the aim of restoring growth deficiency and producing high titers of desired products^9,16,45^.

## Conclusion

Metabolic engineering of *S*. *cerevisiae* was previously proposed to enable microbial production of ginsenosides. The mass production of ginsenosides at an attractive price, along with their widespread usage in clinical applications and the food supplement industry, can be achieved through continuous advances in the engineering of microbial cell factories. In this study, we demonstrated that increasing NADPH availability along with optimization of the transcriptional balance between key enzymes improved PPD production in engineered yeast at flask-level fermentation with minimal media. More than an 11-fold increase in the PPD production titer was achieved from the engineered strain overexpressing ALD6 in place of ALD2 (6.01 mg/L), compared with the initially constructed strain (0.54 mg/L) for PPD production. This improvement was mainly attributed to the increased NADPH/NADP^+^ ratio. Excess NADPH was successfully redirected to PPD production. For further improvement, the *zwf1* gene was deleted to create a biological driving force of carbon-flux redirection toward PPD production. However, this strain coupled with Δ*zwf1* did not increase PPD production, due to the reduction of NADPH availability during the production phase of the growth cycle. The strategy employed in this study illustrates a promising step toward improving strain development for the microbial production of ginsenosides. However, we found that engineering redox balance impaired cell growth, supporting that maintenance of redox cofactors is a critical determinant of cellular growth. Further work will be required to focus on restoring growth defects while ensuring efficient production of PPD through combinatorial engineering strategies including optimization on fermentation process for up-scaled production of PPD. The target genes found to be effective for PPD production could also serve as a basis for further strategies to efficiently produce other terpenoids.

## Methods

### Strains and media

All strains used in this study (Table 1) were constructed in *S*. *cerevisiae* CEN.PK2-1D (*MATα ura3-52 trp1-289 leu2-3*,*112 his3*Δ*1 MAL2-8*^*C*^ *SUC2*) as a parent strain. Yeast strains were cultivated at 30 °C in a yeast synthetic complete (YSC) medium containing 6.7 g/L yeast nitrogen base (Difco), 2% (v/v) glucose, and a mixture of amino acids and nucleotides without uracil (Synthetic Drop-out Medium Supplements, Sigma)^9,28^. *E*. *coli* DH-5α (RBC Real Biotech Co.) was used for the DNA manipulation and plasmid amplification. *E*. *coli* cells were cultivated at 37 °C in Luria-Bertani medium (Difco) with 100 mg/L ampicillin.

### Plasmid construction

All plasmids used in this study and primers used for the construction of the plasmids are listed in Supplementary Tables S1 and S2, respectively. In order to introduce the PPD-producing pathway into the genome of *S*. *cerevisiae*, the sequences of *PgDS*, *PgPPDS*, and *AtCPR1* were codon-optimized and cloned between the *Eco*RI and *Xho*I sites of pUC57-URA3Myc-GPD to produce pUC57-GPD-PgDS, pUC57-GPD-PgPPDS, and pUC57-GPD-AtCPR1, respectively. The codon-optimized *PgDS* and *PgPPDS* were also cloned between the *Eco*RI and *Xho*I sites of pUC57-URA3Myc-CCW12 and pUC57-URA3Myc-ADH2, generating pUC57-CCW-PgDS and pUC57-CCW-PgPPDS, and pUC57-ADH-PgDS and pUC57-ADH-PgPPDS, respectively. The *erg20* gene, encoding farnesyl pyrophosphate synthetase, and the *tHMG1* gene, encoding a truncated HMG-CoA reductase, were cloned between the *Eco*RI and *Xho*I sites of pUC57-URA3Myc-TEF1 and pUC57-URA3Myc-CCW12, generating pUC57-TEF-ERG20 and pUC57-CCW-tHMG1, respectively.

The p416-GPD plasmid, a single copy number plasmid harboring *ura3* as a marker gene and a *GPD* (*TDH3*) promoter, was used as an expression vector for *gnd1*, *gdh2*, *ald6*, *zwf1*, *stb5*, and *zms1* genes. These genes were amplified from *S*. *cerevisiae* CEN.PK2-1D genomic DNA with the primers described in Supplementary Table S1. The *gnd1* and *zwf1* genes were cloned between the *Eco*RI and *Xho*I sites of p416-GPD, generating pGPD-GND1 and pGPD-ZWF1, respectively. The *gdh2* gene was cloned between the *Xba*I and *Sma*I sites of p416-GPD, generating pGPD-GDH2. The *ald6* gene was cloned between the *Bam*HI and *Xho*I sites of p416-GPD, and the *stb5* gene was cloned between the *Eco*RI and *Sal*I sites of p416-GPD, generating pGPD-ALD6 and pGPD-STB5, respectively. The *zms1* gene was cloned between the *Xma*I and *Xho*I sites of p416-GPD, generating pGPD-ZMS1. Finally, the preceding *ald6* and *gdh2* genes were also cloned under the *GPD* promoter of digested pUC57-URA3Myc-GPD, generating pUC57-GPD-ALD6 and pUC57-GPD-GDH2.

### Construction of PPD-producing strains

Standard techniques and media for the genetic modification of *S*. *cerevisiae* were used^46^. Genetic engineering of *S*. *cerevisiae* was performed using the URA3-blaster genetic disruption method^47^. The URA3-blaster method used a recyclable URA3 cassette flanked by 50-bp repeats homologous with the targeted deletion (or insertion) site on yeast’s chromosome. In case of gene integration, a DNA fragment was prepared to include a gene of interest to insert and the URA3-blaster cassette. The homologous flanks were introduced by PCR. Selection for URA prototroph was used to isolate correct transformants carrying the URA3-blaster cassette. After PCR-mediated confirmation of the genetic manipulation on a desired site, counter selection on 5-fluoro-orotic acid identified isolates which have lost the URA3 sequences. Selected URA3-auxotrophic isolates were used for further genetic engineering utilizing the same URA3-blaster method described above. In order to increase the precursor pool for PPD synthesis, the MVA pathway was first modulated. The *leu2* gene was replaced by the *erg20* gene under control of the *TEF1* promoter. The *P*_*TEF1*_-*ERG20* replacement cassette with flanking regions homologous with the *leu2* gene was amplified by PCR from pUC57-TEF-ERG20 and introduced into CEN.PK2-1D. Using the same method described above, the *P*_*CCW12*_-*tHMG1* cassette replacing the *his3* gene was amplified by PCR from pUC57-CCW-tHMG1, and introduced into the CEN.PK2-1D Δ*leu2::P*_*TEF1*_*-ERG20* strain. The *P*_*GPD*_-*AtCPR1* cassette replacing the *lpp1* gene was amplified by PCR from pUC57-GPD-AtCPR1, and introduced into CEN.PK2-1D Δ*leu2::P*_*TEF1*_*-ERG20* Δ*his3::P*_*CCW12*_*-tHMG1*, resulting in strain PPD00 (CEN.PK2-1D Δ*leu2::P*_*TEF1*_*-ERG20* Δ*his3::P*_*CCW12*_*-tHMG1* Δ*lpp1::P*_*GPD*_*-AtCPR1*).

In order to construct PPD-producing *S*. *cerevisiae* strains, PPD00 was further engineered by integrating *PgDS* and *PgPPDS* genes under three different promoters (*P*_*GPD*_, *P*_*CCW12*_, and *P*_*ADH2*_). The *P*_*GPD*_-*PgDS* and *P*_*GPD*_-*PgPPDS* cassettes replacing the genes *dpp1* and *ypl062w*, respectively, were amplified by PCR from pUC57-GPD-PgDS and pUC57-GPD-PgPPDS. The amplified integration cassettes were used to sequentially transform strain PPD00, resulting in strain PPD01 (PPD00 Δ*dpp1::P*_*GPD*_*-PgDS* Δ*ypl062w::P*_*GPD*_*-PgPPDS*).

The *PgDS* and *PgPPDS* genes, under the control of the *CCW12* promoter, were integrated into the same locus of strain PPD00, as described above. The *P*_*CCW12*_-*PgDS* and *P*_*CCW12*_-*PgPPDS* cassettes were amplified by PCR from pUC57-CCW-PgDS and pUC57-CCW-PgPPDS. These amplified cassettes were used to sequentially transform strain PPD00, resulting in strain PPD02 (PPD00 Δ*dpp1::P*_*CCW12*_*-PgDS* Δ*ypl062w::P*_*CCW12*_*-PgPPDS*).

The *PgPPDS* gene under the control of the *ADH2* promoter was integrated into strain PPD00 Δ*dpp1::P*_*CCW12*_*-PgDS* by replacing the *ypl062w* gene. The *P*_*ADH2*_-*PgPPDS* cassette was amplified by PCR from pUC57-ADH-PgPPDS, then used to transform strain PPD00 Δ*dpp1::P*_*CCW12*_*-PgDS*, resulting in strain PPD03 (PPD00 Δ*dpp1::P*_*CCW12*_*-PgDS* Δ*ypl062w::P*_*ADH2*_*-PgPPDS*).

The *PgDS* and *PgPPDS* genes under the control of the *ADH2* and *CCW12* promoters, respectively, were integrated into strain PPD00. The *P*_*ADH2*_-PgDS amplified by PCR from pUC57-ADH-PgDS and *P*_*CCW12*_-*PgPPDS* cassettes were sequentially introduced into strain PPD00, resulting in strain PPD04 (PPD00 Δ*dpp1::P*_*ADH2*_*-PgDS* Δ*ypl062w::P*_*CCW12*_*-PgPPDS*).

The same strategy was used to construct strain PPD05 (PPD00 Δ*dpp1::P*_*ADH2*_*-PgDS* Δ*ypl062w::P*_*ADH2*_*-PgPPDS*). The *PgPPDS* gene under the control of the *ADH2* promoter was integrated into strain PPD00 Δ*dpp1::P*_*ADH2*_*-PgDS* by replacing the *ypl062w* gene. At each step of strain construction, the modifications of genomic DNA were verified by PCR using a pair of primers that flanked the endpoints of the respective target regions.

### Construction of strains with increased NADPH availability

To delete the *ald2* gene, the deletion cassette with flanking regions homologous with the *ald2* gene was amplified by PCR from pUC57-URA3 with a pair of primers, Del_Ald2_F and Del_Ald2_R. The amplified *ald2* gene deletion cassette was introduced into CEN.PK2-1D, resulting in strain −ALD2. The *ald2* gene was also replaced by the *ald6* gene under the control of the *GPD* promoter in the CEN.PK2-1D and PPD05 strains using the above method, resulting in −ALD2 +ALD6 and PPD08, respectively. The *P*_*GPD*_-*ALD6* replacement cassette with flanking regions homologous with the *ald2* gene was amplified by PCR from pUC57-GPD-ALD6 with a pair of primers, Del_Ald2_F and Del_Ald2_R.

The *gdh1* gene was either deleted by introducing the *gdh1* deletion cassette, or replaced by introducing the *P*_*GPD*_*-GDH2* DNA fragment into CEN.PK2-1D, generating strains -GDH1 and -GDH1 + GDH2, respectively. The same method was used to replace the *gdh1* gene of strain PPD05 with the *P*_*GPD*_*-GDH2* fragment, generating strain PPD09.

In order to overexpress the *gnd1*, *gdh2*, or *ald6* gene in CEN.PK2-1D, the plasmid pGPD-GND1, pGPD-GDH2, or pGPD-ALD6 was transformed into CEN.PK2-1D, resulting in strains +GND1, +GDH2, and +ALD6, respectively. To overexpress the *zwf1* or *stb5* gene in CEN.PK2-1D and PPD05, the plasmid pGPD-ZWF1 or pGPD-STB5 was transformed into the CEN.PK2-1D and PPD05 strains, resulting in +ZWF1, PPD06, +STB5 and PPD07, respectively. To overexpress the *zms1* gene in the PPD05 and PPD08 strains, the plasmid pGPD-ZMS1 was transformed into PPD05 and PPD08, resulting in PPD10 and PPD11, respectively.

To further modulate the NADPH generating flux, the *zwf1* gene was deleted by introducing the *zwf1* gene deletion cassette into PPD08, resulting in strain PPD12.

### PPD production by flask fermentation

YSC medium with 2% (v/v) glucose, lacking uracil, was used to cultivate yeast strains for PPD production. The PPD-producing strains were grown on YSC agar plates with 2% (v/v) glucose, lacking uracil, and then transferred to 50 mL conical tubes containing 10 mL of seed medium. After overnight cultivation at 30 °C with 250 rpm, 250 mL flasks containing 50 mL medium were inoculated with these seed cultures to give an initial OD_600_ of 0.5. The flask fermentation was run at 30 °C with 250 rpm for 6 days. All the flask fermentations were performed in three independent experiments.

### Extraction and analytical methods

All optical densities at 600 nm (OD_600_) were measured using a Shimadzu UV-2600 spectrophotometer. Yeast cells were collected by centrifugation at 13,000 × *g* for 5 min to contain an equivalent OD_600_ of 20. The collected cells were then resuspended in 0.6 mL of a methanol-acetone (MA) solution mixed in the same ratio, before being lysed by a FastPrep-24 5 G homogenizer (MP Biomedicals, California, USA) according to the manufacturer’s directions. After filtration using 0.2 µm syringe filters, the metabolites secreted in the MA solution were analyzed by an Agilent high performance liquid chromatography (HPLC) system equipped with a UV detector at 203 nm. Samples were separated on a Kinetex 5 µm EVO C18 column (150 mm × 4.6 mm; Phenomenex, Aschaffenburg, Germany) at 30 °C with an isocratic elution of 1 mL/min flow rate for 30 min. The mobile phase consisted of acetonitrile, methanol, and water in a ratio of 90:9:1 (v/v).

Intracellular concentrations of NADPH and NADP^+^ were quantified using the EnzyChrom NADP^+^/NADPH assay kit (Cat. No. ECNP-100; Bioassay Systems, Hayward, CA). Cells were harvested by centrifugation and resuspended in cold PBS buffer solution to an OD_600_ of 10. Samples were treated with the NADPH or NADP^+^ extraction buffers and all subsequent steps were performed according to the manufacturer’s instructions.

### Statistical analysis

Probability analysis to determine statistical significance was performed using the Student’s *t*-test with a two-tailed distribution. Comparing to the appropriate control strain, *p*-values less than 0.05 (*P* < 0.05) were considered to be significant for this study.

## Electronic supplementary material

Supplementary information
