# Supplementary material for: Rerouting of NADPH synthetic pathways for increased protopanaxadiol production in Saccharomyces cerevisiae
Source: Sci Rep. 2018 Oct 25;8:15820. doi: 10.1038/s41598-018-34210-3 (PMC6202386; doi:10.1038/s41598-018-34210-3)
Supplement: Supplementary file 1 — Supplementary information [file 41598_2018_34210_MOESM1_ESM.docx]

**Supplementary information**

**Rerouting of NADPH synthetic pathways for increased protopanaxadiol production in *Saccharomyces cerevisiae***

Jae-Eung Kim^1^, In-Seung Jang^1^, Bong Hyun Sung^2^, Sun Chang Kim^3^* and Ju Young Lee ^1^*

^1^ Center for Bio-based Chemistry, Korea Research Institute of Chemical Technology (KRICT), 406-30, Jongga-ro, Jung-gu, Ulsan, 44429, Republic of Korea

^2^ Cell Factory Research Center, Korea Research Institute of Bioscience and Biotechnology (KRIBB), Daejeon, 34141, Republic of Korea

^3^ Department of Biological Sciences, Korea Advanced Institute of Science and Technology (KAIST), Daejeon, 34141, Republic of Korea

Corresponding authors

*S.C.K.

Phone: +82-42-350-2619

Fax: +82-42-350-2610

E-mail: sunkim@kaist.ac.kr

*J.Y.L.

Phone: +82-52-241-6325

Fax: +82-52-241-6359

E-mail: juylee@krict.re.kr

# Table S1. List of primers used in this study.

| **Primer name** | **Primer sequence (5’-3’)** | **Application** |
| --- | --- | --- |
| Gnd1_F | ggaattcatgtctgctgatttcggttt | Amplifying the *gnd1* gene from gDNA of *Saccharomyces cerevisiae* |
| Gnd1_R | ccgctcgagttaagcttggtatgtagaggaagaa |  |
| Gdh2_F | gctctagaatgctttttgataacaaaaatcgcgg | Amplifying the *gdh2* gene from gDNA of *Saccharomyces cerevisiae* |
| Gdh2_R | tcccccgggtcaagcacttgcctccgctt |  |
| Ald6_F | cgggatccatgactaagctacactttgacactgc | Amplifying the *ald6* gene from gDNA of *Saccharomyces cerevisiae* |
| Ald6_R | ccgctcgagttacaacttaattctgacagcttttacttcag |  |
| Zwf1_F | ggaattcatgagtgaaggccccgtcaa | Amplifying the *zwf1* gene from gDNA of *Saccharomyces cerevisiae* |
| Zwf1_R | ccgctcgagctaattatccttcgtatcttctggc |  |
| Stb5_F | ggaattcatggatggtcccaattttgcac | Amplifying the *stb5* gene from gDNA of *Saccharomyces cerevisiae* |
| Stb5_R | acgcgtcgactcatacaagtttatcaacccaagagacg |  |
| Del_Ald2_F | ttacattgcatgtccatcaaaaacaatcgtgaaaataagccaaaagaaaaccagtcacgacgttgtaaaa | Amplifying a deletion /replacement cassette integrating into *ald2* locus |
| Del_Ald2_R | ctgcaacatcccactccttctttgcagtttctttaaacttttcaacaaacaggtttcccgactggaaagc |  |
| Del_Gdh1_F | actatcgcattattctaatataacagttaggagaccaaaaagaaaaagaaccagtcacgacgttgtaaaa | Amplifying a deletion /replacement cassette integrating into *gdh1* locus |
| Del_Gdh1_R | gacggcaatagcttctggagtggaacccatgttggaaccttcggcaataaaggtttcccgactggaaagc |  |
| Del_Zwf1_F | tatagacagaaagagtaaatccaatagaatagaaaaccacataaggcaagccagtcacgacgttgtaaaa | Amplifying a deletion cassette integrating into *zwf1* locus |
| Del_Zwf1_R | cctcccaacgctcgttttcgatgttgaaagtcattgctgcaaaagtgacaaggtttcccgactggaaagc |  |

#

# Table S2. List of plasmids used in this study.

| **Plasmids** | **Description** | **Reference** |
| --- | --- | --- |
| pUC57-URA3Myc-GPD | pUC57-based cloning vector containing *GPD* promoter and ampicillin resistance gene (*bla*) | ([Lee et al., 2015](#_ENREF_1)) |
| pUC57-URA3Myc-CCW12 | pUC57-based cloning vector containing *CCW12* promoter and ampicillin resistance gene (*bla*) | ([Lee et al., 2015](#_ENREF_1)) |
| pUC57-URA3Myc-ADH2 | pUC57-based cloning vector containing *ADH2* promoter and ampicillin resistance gene (*bla*) | ([Lee et al., 2015](#_ENREF_1)) |
| pUC57-URA3Myc-TEF1 | pUC57-based cloning vector containing *TEF1* promoter and ampicillin resistance gene (*bla*) | ([Lee et al., 2015](#_ENREF_1)) |
| pUC57-URA3 | pUC57-based deletion vector containing ampicillin resistance gene (*bla*) | ([Lee et al., 2015](#_ENREF_1)) |
| pUC57-TEF-ERG20 | pUC57-URA3Myc-TEF1 harboring ERG20 | This study |
| pUC57-CCW-tHMG1 | pUC57-URA3Myc-CCW12 harboring tHMG1 | This study |
| pUC57-GPD-AtCPR | pUC57-URA3Myc-GPD harboring AtCPR1 | This study |
| pUC57-GPD-PgDS | pUC57-URA3Myc-GPD harboring PgDS | This study |
| pUC57-GPD-PgPPDS | pUC57-URA3Myc-GPD harboring PgPPDS | This study |
| pUC57-CCW-PgDS | pUC57-URA3Myc-CCW12 harboring PgDS | This study |
| pUC57-CCW-PgPPDS | pUC57-URA3Myc-CCW12 harboring PgPPDS | This study |
| pUC57-ADH-PgDS | pUC57-URA3Myc-ADH2 harboring PgDS | This study |
| pUC57-ADH-PgPPDS | pUC57-URA3Myc-ADH2 harboring PgPPDS | This study |
| pUC57-GPD-ALD6 | pUC57-URA3Myc-GPD harboring ALD6 | This study |
| pUC57-GPD-GDH2 | pUC57-URA3Myc-GPD harboring GDH2 | This study |
| p416-GPD | Expression vector containing *GPD* promoter, CEN/ARS single-copy origin and URA3 marker | ([Lee et al., 2015](#_ENREF_1)) |
| pGPD-GND1 | GND1 expression, in p416-GPD | This study |
| pGPD-GDH2 | GDH2 expression, in p416-GPD | This study |
| pGPD-ALD6 | ALD6 expression, in p416-GPD | This study |
| pGPD-ZWF1 | ZWF1 expression, in p416-GPD | This study |
| pGPD-STB5 | STB5 expression, in p416-GPD | This study |
| pGPD-ZMS1 | ZMS1 expression, in p416-GPD | This study |

**References**

Lee, J. Y., Kang, C. D., Lee, S. H., Park, Y. K., Cho, K. M., 2015. Engineering cellular redox balance in *Saccharomyces cerevisiae* for improved production of L‐lactic acid. Biotechnol. Bioeng. 112**,** 751-758.
